# How Motivation Triggers Speedy Decisions

**DOI:** 10.1371/journal.pbio.1001812

**Published:** 2014-03-18

**Authors:** Janelle Weaver

**Affiliations:** Freelance Science Writer, Carbondale, Colorado, United States of America

**Figure:**
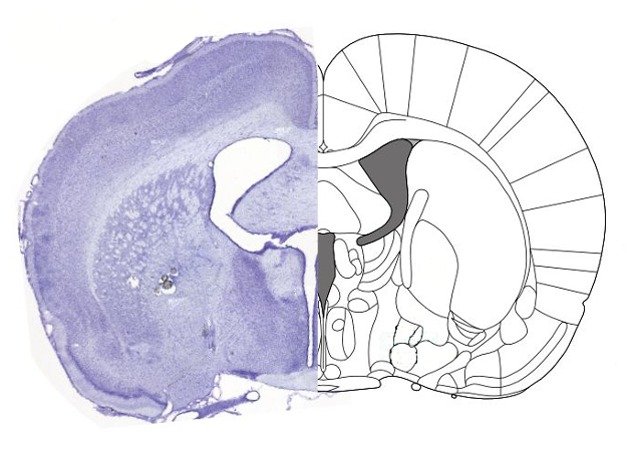
Avila and Lin report that electrical stimulation of the basal forebrain in rats increased decision-making speed.

Animals must react quickly to objects and events in the environment to survive, especially when their decisions could result in a reward or punishment. Based on this fact, scientists have assumed that the speed of decision-making during behavioral tasks is affected by motivational salience—the extent to which an object or event predicts important behavioral outcomes. Neurons in a brain region called the basal forebrain (BF) respond to motivationally salient stimuli, but the influence of these BF neurons on decision-making speed has been unclear.

In a study published this month in *PLOS Biology*, Irene Avila and Shih-Chieh Lin of the National Institute on Aging at the National Institutes of Health provide new insights into how motivational salience not only speeds up reaction times but also reduces variability in decision-making speed. Avila and Lin's findings suggest that the activity of BF neurons determines the speed of rats' decisions in response to motivationally salient stimuli, providing a possible neural explanation for the slower decision-making speeds seen in conditions ranging from depression to dementia.

To examine the relationship between motivational salience and decision-making speed, Avila and Lin trained rats to stick their nose through a port in a Plexiglas chamber and wait for a noise that signaled a reward. White noise indicated that the rats would receive a large reward of four drops of water, whereas a clicking sound signaled a small reward of only one drop of water. During some trials, no noise or reward was presented.

Based on this auditory information, the rats decided whether to approach a nearby location in the chamber to receive the water reward. To quantify decision-making speed, the researchers timed how long it took for the rats to remove their nose from the port after hearing a sound. The rats almost always moved toward the reward location following white noise or a clicking sound but rarely did so when neither of these sounds was broadcast. Moreover, their reaction times were faster when the sound indicated a large reward (white noise) compared with a small reward (clicking).

Neural recordings during the task revealed that BF neurons responded more strongly to sounds that signaled a large reward compared with sounds signaling a small reward, confirming that the activity of these neurons is affected by motivational salience. Moreover, the activity of BF neurons predicted rats' decision-making speed. The rats' BF neurons began to fire about 50 milliseconds after the onset of the sound and showed peak activity about 70 milliseconds later, long before the rats removed their nose from the chamber port. This finding suggests that the activity of BF neurons occurred early enough to affect decision-making speed.

To examine the causal relationship between neural activity and decision-making speed, the researchers electrically stimulated BF neurons shortly after broadcasting a tone that signaled a reward. The greater the electrical stimulation, the more quickly the rats responded to the tone. This finding indicates that BF neurons play a major role in determining the speed of decisions.

Slow reaction times are a key feature of a range of psychiatric and neurological conditions, including depression, schizophrenia, dementia, and cognitive aging. Avila and Lin's findings offer a possible explanation of the neural basis of this phenomenon, revealing a potentially important role for a previously neglected population of neurons. Future research on the properties of these neurons could shed new light on potential treatment strategies for disorders associated with slow decision-making.

**Avila I, Lin S-C (2014) Motivational Salience Signal in the Basal Forebrain Is Coupled with Faster and More Precise Decision Speed.** doi:10.1371/journal.pbio.1001811

